# Author Correction: Potential of artificial intelligence in reducing energy and carbon emissions of commercial buildings at scale

**DOI:** 10.1038/s41467-025-63487-y

**Published:** 2025-08-29

**Authors:** Chao Ding, Jing Ke, Mark Levine, Jessica Granderson, Nan Zhou

**Affiliations:** Energy Technologies Area, Lawrence Berkeley National Laboratory, One Cyclotron Road, Berkeley, CA 94720 USA

**Keywords:** Energy modelling, Energy efficiency

Correction to: *Nature Communications*; 10.1038/s41467-024-50088-4, published online 14 July 2024

In the version of the article initially published, Jessica Granderson (Energy Technologies Area, Lawrence Berkeley National Laboratory, Berkeley, CA, USA) was not included in the author list and now appears in the HTML and PDF versions of the article.

