# Supplementary material for: Potential of artificial intelligence in reducing energy and carbon emissions of commercial buildings at scale
Source: Nat Commun. 2024 Jul 14;15:5916. doi: 10.1038/s41467-024-50088-4 (PMC11247084; doi:10.1038/s41467-024-50088-4)
Supplement: Supplementary file 1 — Supplementary Information [file 41467_2024_50088_MOESM1_ESM.pdf]

Potential of Artificial Intelligence in Reducing Energy and Carbon Emissions of Commercial Buildings at Scale

Chao Ding, Jing Ke, Mark Levine, Jessica Granderson, Nan Zhou*

Energy Technologies Area, Lawrence Berkeley National Laboratory, One Cyclotron Road, Berkeley, CA 94720, USA

Corresponding author: Nan Zhou, nzhou@lbl.gov

Supplementary information

The building energy consumption and carbon emission data generated in this study are available at https://doi.org/10.6084/m9.figshare.26043604.

Supplementary Figures

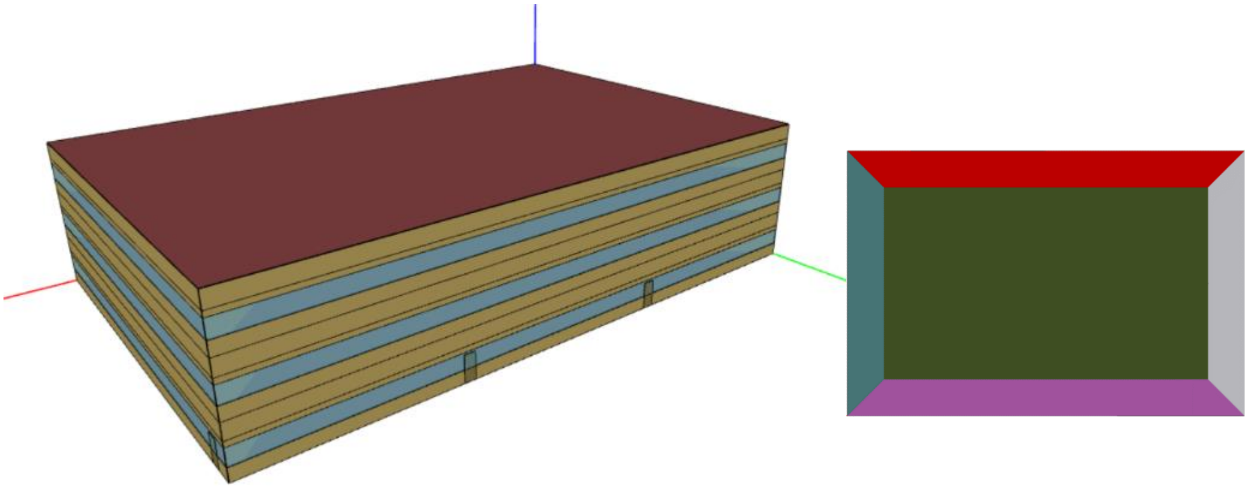

Supplementary Figure 1 | Geometry and the thermal zoning of the medium office building model.

Supplementary Tables

Supplementary Table 1 | Baseline EnergyPlus model settings

Input parameters	Unit	Medium office building prototype
External wall insulation	W/m²K	0.48
Roof insulation	W/m²K	0.36
Ground floor insulation	W/m²K	2.14
External windows	W/m²K	3.05
Infiltration rate	$m^3/s/m^2$	0.001 $m^3/s/m^2$, door infiltration = 1.04 m^3/s
Lighting power density	W/m^2	10.76
Plug load power density	W/m^2	8.07
Occupancy density	m^2/ person	18.58
HVAC system	-	Packaged air conditioning unit, VAV terminal box with damper and electric reheating coil
Thermostat setpoint	°C	24°F cooling / 21°F heating

Supplementary Table 2 | Energy saving potential from equipment efficiency improvement

Case	Description	Honolulu (1A)		Los Angeles (3B)		Baltimore (4A)		Buffalo (5A)	
		kWh/m^2	% improvement	kWh/m^2	% improvement	kWh/m^2	% improvement	kWh/m^2	% improvement
1	20% cooling efficiency improvement	152	6.0	131	0.3	151	2.6	165	1.4
2	12% heating efficiency improvement	162	0	132	0.0	154	0.8	165	1.4
3	Combine Cases 1 and 2	152	6.0	131	0.3	150	3.4	163	2.8
4	15% LPD reduction	153	5.5	124	6.0	149	4.2	162	3.6
5	21% LPD reduction	150	7.0	121	7.8	147	5.4	160	4.6
6	10% EPD reduction	155	0.1	126	4.3	151	2.9	164	2.3
7	20% EPD reduction	149	4.3	121	8.3	147	5.5	161	4.1
8	Integrate Cases 3, 5, and 7	128	17.3	110	16.1	134	14.0	148	11.5
9	Integrate Cases 3, 5, and 7+HP for space heating[1]	128	17.3	110	16.2	125	19.6	132	21.5

Note: LPD stands for lighting power density; EPD stands for equipment power density; HP stands for heat pump.[1.] The range of annual effective heating system COP is 2.1~5.6 varying by states and climates (CaraDonna et al., 2023)

Supplementary Table 3 | Energy saving potential from building design and construction

Scenario	Description	Honolulu (1A)		Los Angeles (3B)		Baltimore (4A)		Buffalo (5A)	
		kWh/m^2	% improve-ment	kWh/m^2	% improve-ment	kWh/m^2	% improve-ment	kWh/m^2	% improve-ment
1	East Orientation	164	-1.3	135	-2.4	158	-1.4	169	-0.6
2	South Orientation	162	-0.0	132	-0.1	156	-0.1	168	-0.0
3	West Orientation	164	-1.3	135	-2.4	158	-1.4	169	-0.6
4	High Insulation	156	3.2	123	6.7	148	4.8	158	5.9
5	Low Infiltration	161	0.3	131	0.3	153	1.4	163	2.8
6	WWR = 0.2	156	3.5	124	5.7	151	3.0	162	3.3
7	WWR = 0.4	165	-2.2	133	-0.7	158	-1.9	171	-1.9
8	WWR = 0.6	175	-8.0	142	-7.7	167	-7.7	182	-8.5
9	Integrate Scenarios 4 and 6	152	5.9	120	9.1	142	8.7	153	8.7

Note: WWR stands for window to wall ratio. The baseline orientation is north. The other three orientations are achieved through rotating the building by 90° (east), 180° (south), and 270° (west).

Supplementary Table 4 | U-factors of the high insulation case

Envelop U factor [W/m^2-K]	Opaque wall	Roof	Window
Baseline	0.48	0.36	3.05
High insulation	0.31	0.18	2.25

Supplementary Table 5 | Scenario definitions.

Scenario	Description
Frozen (FRO)	A scenario where the market shares of three types of buildings (baseline buildings, high energy efficiency buildings, and net zero energy buildings) remain constant at the 2020 level throughout the future until 2050.
BAU wo/ AI	Business as usual scenario without AI's contribution. This scenario projects that the market shares of high energy efficiency buildings and net zero energy buildings will increase at a lower rate without energy policy.
BAU w/ AI*	Business as usual scenario with AI's contribution.
Policy wo/ AI**	Policy scenario without AI's contribution. This scenario projects that the market shares of high energy efficiency buildings and net zero energy buildings will increase at a higher rate with energy policy.
Policy w/ AI**	Policy scenario with AI's contribution.
Policy w/ AI & LEPG***	Policy scenario with AI's contribution and LEPG.

* AI is assumed to contribute an additional 10% reduction on average in the cost premium of HEE/NZE buildings.

** The policy scenario includes building efficiency programs, incentives, rebates, or subsides that can further reduce the cost premium of HEE/NZE buildings.

*** LEPG stands for low emission power generation (decreasing from the 2020 level to zero emission by 2050).

Supplementary Table 6 | Projected energy use by scenario*

Scenario	2020	2030	2040	2050
Frozen (FRO)	155	170	187	203
BAU wo/ AI	155	169	179	174
BAU w/ AI	155	168	170	160
Policy wo/ AI*	155	167	161	124
Policy w/ AI*	155	164	145	100
Policy w/ AI & LEPG*	155	164	145	100

*Average projected energy use (million MMBTU) from sensitivity analysis.

Supplementary Table 7 | Projected CO_2 emissions by scenario*

Scenario	2020	2030	2040	2050
Frozen (FRO)	155	170	187	203
BAU wo/ AI	155	169	179	174
BAU w/ AI	155	168	170	160
Policy wo/ AI*	155	167	161	124
Policy w/ AI*	155	164	145	100
Policy w/ AI & LEPG*	155	164	145	100

* Average projected CO_2 emissions (million metric tons) from sensitivity analysis.

Supplementary Table 8 | Cost premium for high energy-efficiency (HEE) building and net zero energy (NZE) building

	High energy-efficiency building (ECMs) (%)	Net zero energy (Renewables with ECMs) (%)[35]
Medium office new construction	6	10
Medium office deep renovation	12	19

Note: ECM stands for energy conservation measure.

Supplementary Table 9 | Construction cost premium for new HEEBs and NZEBs in 2020 by climate zones (CZ)

Building Type	Cost ($/sf)	CZ 5A	CZ 4A	CZ 1A	CZ 3B (CA)
Baseline building	Construction cost	167	134	122	158
	Development cost	57	45	41	53
	Total cost	224	179	163	211
HEE building	Δ Construction cost	10	8	7	9
	Δ Development cost	3	3	3	3
	Δ Total cost	13	10	10	13
NZE building	Δ Construction cost	17	13	12	16
	Δ Development cost	5	5	4	5
	Δ Total cost	22	18	16	21

Note: Δ denotes cost increase from the baseline building.[1] The selected climate zones (CZs) represent the top four regions (Honolulu: CZ 1A, Los Angeles: CZ 3B, Baltimore: CZ 4A, and Buffalo: CZ 5A) defined by the International Energy Conservation Code (IECC) based on number of office buildings from CBECS 2012 data.[2,3] Construction cost and development cost were estimated by authors based on RSMeans construction costs data.[4]

Supplementary Table 10 | Deep retrofit cost for existing medium office buildings in 2020 by climate zones (CZ)

Building Type	Cost ($/sf)	CZ 5A	CZ 4A	CZ 1A	CZ 3B (CA)
Baseline building	Construction cost	68	52	47	65
	Development cost	32	24	22	31
	Total cost	101	76	70	95
HEE building	Δ Construction cost	9	6	6	7
	Δ Development cost	4	3	3	3
	Δ Total cost	12	9	8	12
NZE building	Δ Construction cost	13	10	9	12
	Δ Development cost	6	5	5	5
	Δ Total cost	19	15	13	18

Note: Δ denotes cost increase from the baseline building.[1] The selected climate zones (CZs) represent the top four regions (Honolulu: CZ 1A, Los Angeles: CZ 3B, Baltimore: CZ 4A, and Buffalo: CZ 5A) defined by the International Energy Conservation Code (IECC) based on number of office buildings from CBECS 2012 data.[2,3] Construction cost and development cost were estimated by authors based on RSMeans construction costs data.[4]

Supplementary Table 11 | Decrease in cost premium for HEE/NZE medium office buildings

Scenario	2020 (%)	2030 (%)	2040 (%)	2050 (%)
Frozen (FRO)	0	0	0	0
BAU wo/ AI	0	20	40	60
BAU w/ AI*	0	30	50	70
Policy wo/ AI**	0	40	60	80
Policy w/ AI**	0	50	70	90
Policy w/ AI & LEPG***	0	50	70	90

* AI is assumed to contribute an additional 10% reduction on average in the cost premium of HEE/NZE buildings.

** The policy scenario includes building efficiency programs, incentives, rebates, or subsides that can further reduce the cost premium of HEE/NZE buildings.

*** LEPG stands for low emission power generation (decreasing from the 2020 level to zero emission by 2050).

Supplementary Table 12 | Annual retrofit share of total surviving medium office floor space

Scenario	2020 (%)	2030 (%)	2040 (%)	2050 (%)
Frozen (FRO)	0	0	0	0
BAU wo/ AI	0.5	0.5	0.5	0.5
BAU w/ AI	0.5	0.5	0.5	0.5
Policy wo/ AI*	0.5	1.5	2.5	3.5
Policy w/ AI*	0.5	1.5	2.5	3.5
Policy w/ AI & LEPG*	0.5	1.5	2.5	3.5

Note: Annual retrofit shares were estimated by Authors. The policy scenario includes retrofit programs, incentives, rebates, or subsides that can increase the retrofit share of existing buildings.

Supplementary Table 13 | Maximum allowed NZEB share of the new medium office*

Scenario	2020 (%)	2030 (%)	2040 (%)	2050 (%)
BAU wo/ AI	57	57	57	57
BAU w/ AI**	59	59	59	59
Policy wo/ AI***	77	77	77	77
Policy w/ AI***	79	79	79	79
Policy w/ AI & LEPG***	79	79	79	79

* Maximum allowed NZEB shares were estimated by authors based on Kurdgelashvili et al.[5] and CBECS[2]. Once the share of NZEB reaches the maximum value, it will not increase further.

** AI could help reduce the energy use, improve energy management, and optimize energy generation and storage, and therefore could increase the share (assuming by 2%) of buildings that be built as NZEB.

*** Policy scenario includes incentives, rebates or subsides, more energy-efficient technologies, and programs that can further increase the share of NZEB, even it may be difficult and not cost-effective to reach this high share.

Supplementary Table 14 | Maximum allowed NZEB share of the retrofitted medium office*

Scenario	2020 (%)	2030 (%)	2040 (%)	2050 (%)
BAU wo/ AI	16	16	16	16
BAU w/ AI**	18	18	18	18
Policy wo/ AI***	44	44	44	44
Policy w/ AI***	46	46	46	46
Policy w/ AI & LEPG***	46	46	46	46

* Maximum allowed NZEB shares were estimated by authors based on Kurdgelashvili et al.[5] and CBECS[2]. Once the share of NZEB reaches the maximum value, it will not increase further.

** AI could help reduce the energy use, improve energy management, and optimize energy generation and storage, and therefore could increase the share (assuming by 2%) of buildings retrofitted to NZEB.

*** The policy scenario includes incentives, rebates or subsides, more energy-efficient technologies, and programs that can further increase the share of NZEBs, even it may be difficult and not cost-effective to reach this high share.

Supplementary References

1 NBI. Net zero and living building challenge financial study: a cost comparison report for buildings. (New Building Institute, 2014).

2 US EIA. *Commercial Building Energy Consumption Survey (CBECS)*, <https://www.eia.gov/consumption/commercial/data/2012/> (2012).

3 IECC. *2012 international energy conservation code*, <https://codes.iccsafe.org/content/IECC2012> (2012).

4 Gordian. RSMeans construction costs data. (2022).

5 Kurdgelashvili, L., Li, J., Shih, C.-H. & Attia, B. Estimating technical potential for rooftop photovoltaics in California, Arizona and New Jersey. *Renewable Energy* **95**, 286-302, doi:https://doi.org/10.1016/j.renene.2016.03.105 (2016).
